# Attention-Based Transfer Enhancement Network for Cross-Corpus EEG Emotion Recognition

**DOI:** 10.3390/s25185718

**Published:** 2025-09-13

**Authors:** Zongni Li, Kin-Yeung Wong, Chan-Tong Lam

**Affiliations:** 1 Faculty of Applied Sciences, Macao Polytechnic University, Macao 999078, China; p2109651@mpu.edu.mo (Z.L.); ctlam@mpu.edu.mo (C.-T.L.); 2 Guangxi Key Laboratory of Machine Vision and Intelligent Control, Wuzhou University, Wuzhou 543000, China

**Keywords:** cross-corpus, emotion recognition, domain adaptation, self-supervised learning

## Abstract

A critical challenge in EEG-based emotion recognition is the poor generalization of models across different datasets due to significant domain shifts. Traditional methods struggle because they either overfit to source-domain characteristics or fail to bridge large discrepancies between datasets. To address this, we propose the Cross-corpus Attention-based Transfer Enhancement network (CATE), a novel two-stage framework. The core novelty of CATE lies in its dual-view self-supervised pre-training strategy, which learns robust, domain-invariant representations by approaching the problem from two complementary perspectives. Unlike single-view models that capture an incomplete picture, our framework synergistically combines: (1) Noise-Enhanced Representation Modeling (NERM), which builds resilience to domain-specific artifacts and noise, and (2) Wavelet Transform Representation Modeling (WTRM), which captures the essential, multi-scale spectral patterns fundamental to emotion. This dual approach moves beyond the brittle assumptions of traditional domain adaptation, which often fails when domains are too dissimilar. In the second stage, a supervised fine-tuning process adapts these powerful features for classification using attention-based mechanisms. Extensive experiments on six transfer tasks across the SEED, SEED-IV, and SEED-V datasets demonstrate that CATE establishes a new state-of-the-art, achieving accuracies from 68.01% to 81.65% and outperforming prior methods by up to 15.65 percentage points. By effectively learning transferable features from these distinct, synergistic views, CATE provides a robust framework that significantly advances the practical applicability of cross-corpus EEG emotion recognition.

## 1. Introduction

Emotion recognition has become a cornerstone of affective computing and human–computer interaction, with profound implications for mental health monitoring, adaptive user interfaces, and clinical diagnosis [1,2]. Among various physiological signals, electroencephalography (EEG) offers a direct window into the brain’s emotional processing. Its high temporal resolution and objective nature make it an ideal modality for capturing genuine emotional states, as EEG signals reflect neural activity that is difficult to consciously manipulate [3]. Advances in deep learning have enabled models to achieve impressive accuracies, often exceeding 95%, on single, standardized datasets like DEAP and SJTU Emotion EEG Dataset (SEED) [4,5].

However, this success in controlled settings masks a critical challenge that severely impedes real-world application: the poor generalization of models across different datasets, a problem known as the cross-corpus generalization challenge [6,7]. When a model trained on one dataset is tested on another, its performance often plummets by 20–30% [8,9]. This performance degradation arises from significant “domain shifts” caused by a combination of factors: inter-subject physiological variability (e.g., brain anatomy, skull thickness) and inter-dataset discrepancies in experimental protocols, emotion elicitation stimuli, recording equipment, and ambient noise [3,10]. This gap between laboratory performance and practical applicability remains the primary hurdle for deploying robust EEG-based emotion recognition systems.

To bridge this domain gap, researchers have explored a variety of computational strategies. Early attempts relied on conventional supervised learning, which failed to generalize due to overfitting to source-specific features. Subsequently, domain adaptation techniques [11], such as Domain Adversarial Neural Networks, were introduced to align feature distributions between source and target domains [5]. However, these methods often presuppose a closer proximity between domain distributions than typically exists in diverse EEG datasets, limiting their effectiveness. More recent efforts have incorporated advanced deep learning architectures. Attention mechanisms, particularly multi-head self-attention from Transformers, have shown promise in adaptively focusing on the most informative EEG channels and temporal segments [4,9]. Concurrently, self-supervised and contrastive learning paradigms have emerged as a powerful means to learn subject-invariant representations from unlabeled data, reducing the costly dependency on manual annotation [3,12]. These methods learn robust features by leveraging the inherent structure of EEG signals, for instance, by predicting temporal context or contrasting different augmentations of the same sample.

Despite these advancements, existing methods still exhibit significant limitations when faced with the complexities of cross-corpus EEG data. Each approach, while addressing a piece of the puzzle, fails to provide a comprehensive solution. The primary shortcomings of current state-of-the-art approaches are systematically summarized in Table 1.

Traditional domain adaptation methods like DANN assume that source and target distributions can be aligned through adversarial training. However, this assumption breaks down in cross-corpus EEG scenarios due to: (1) hardware variations—different EEG systems use varying sampling rates, electrode materials, and amplifier characteristics, creating systematic differences in signal properties; (2) protocol differences—SEED uses movie clips while other datasets may use images or music, resulting in fundamentally different neural response patterns; (3) subject heterogeneity—age, cultural background, and individual brain anatomy create irreducible inter-subject variability. When these differences accumulate, forcing alignment between such disparate distributions can cause negative transfer, where the model learns spurious correlations that harm performance. To systematically address these multifaceted challenges identified in Table 1, our proposed CATE framework introduces targeted solutions: (1) against overfitting in supervised learning, CATE employs two-stage training with self-supervised pre-training to learn generalizable features rather than memorizing source-specific patterns; (2) against domain adaptation’s distribution assumptions, CATE’s dual-view strategy learns inherently robust representations without forced alignment, where NERM models domain variations as noise while WTRM extracts domain-invariant multi-scale patterns; (3) against single-view limitations, CATE’s dual-view architecture simultaneously captures complementary information with NERM providing frequency-domain robustness and WTRM capturing temporal-frequency dynamics through wavelet decomposition; and (4) against augmentation dependency, CATE introduces EEG-specific strategies with NERM’s adaptive noise injection and WTRM’s wavelet-based augmentation that preserve emotional content while ensuring robust feature learning. This comprehensive design enables CATE to effectively overcome the limitations of existing approaches.

Several recent architectures have attempted to address these limitations through more sophisticated self-supervised and contrastive learning approaches. Generic contrastive methods adapted for EEG, including SimCLR [13] and Mixup variants [14], employ augmentation strategies unsuited for EEG’s non-stationary nature and operate on single-view representations, missing complementary multi-domain information. GMSS [15] applies SimCLR-based contrastive learning with meiosis augmentation but focuses solely on temporal features, neglecting crucial spatial channel interactions. MV-SSTMA [16] employs masked autoencoding across multiple domains but shows significant performance degradation in self-supervised settings. JCFA [17] uses dual-view contrastive learning from time-frequency domains and incorporates graph convolution during fine-tuning to model channel relationships; however, it suffers from prohibitively long training times due to the computational complexity of graph operations and lacks explicit mechanisms for handling domain-specific noise while assuming similar cross-domain distributions—an assumption that often fails in practice.

These limitations motivate our CATE framework, which introduces a fundamentally different approach to domain adaptation. Unlike existing methods that attempt to force alignment between disparate domains or rely on computationally expensive graph structures, CATE addresses domain shift through two synergistic mechanisms: (1) Noise-Enhanced Representation Modeling (NERM) actively learns to filter out domain-specific artifacts by training on adaptively noise-perturbed data, forcing the model to focus on robust, emotion-relevant patterns rather than superficial recording characteristics; and (2) Wavelet Transform Representation Modeling (WTRM) captures domain-invariant differential entropy patterns across frequency bands that persist across different recording conditions and protocols. This dual-view architecture, combined with efficient attention-based feature fusion instead of graph convolution, enables CATE to achieve superior cross-corpus performance with significantly reduced computational overhead compared to existing approaches. To address these multifaceted challenges, this paper introduces a novel Cross-corpus Attention-based Transfer Enhancement network (CATE). Our approach is built on a comprehensive two-stage framework that synergistically combines self-supervised pre-training with supervised fine-tuning. The core of CATE is a dual-view pre-training strategy that learns complementary representations from two perspectives: (1) NERM, which builds robustness to domain-specific noise and artifacts, and (2) Wavelet Transform Representation Modeling (WTRM), which captures essential frequency-domain differential entropy characteristics of EEG signals. During fine-tuning, attention mechanisms fuse these complementary features to perform robust emotion classification. A multi-component loss function, integrating alignment, contrastive, and style diversity objectives, ensures that the learned features are consistent, discriminative, and generalizable.

This work makes several key contributions to cross-corpus EEG-based emotion recognition:

- We propose a novel dual-view self-supervised pre-training strategy that comprehensively captures complementary signal characteristics—noise robustness and multi-scale frequency patterns—via the NERM and WTRM views, respectively.
- We design a multi-component optimization objective that effectively balances feature consistency, discriminability, and diversity, providing a solid foundation for cross-corpus generalization.
- We develop an attention-based transfer enhancement mechanism that dynamically fuses multi-scale features while maintaining computational efficiency.
- Through extensive experiments on the SEED series datasets, we demonstrate that CATE achieves state-of-the-art performance on six cross-corpus tasks, with accuracy improvements of 5.42% to 15.65% over existing methods, thereby setting a new benchmark for robust, practical EEG-based emotion recognition.
- We pioneer the comprehensive evaluation of cross-corpus emotion recognition across multiple SEED datasets (SEED, SEED-IV, and SEED-V), particularly introducing SEED-V with its challenging five-class emotion taxonomy into cross-corpus research for the first time. Through extensive experimentation comparing multiple state-of-the-art models on these diverse emotion classification tasks (3-class, 4-class, and 5-class), we establish new benchmarks for multi-class cross-corpus EEG emotion recognition and demonstrate the scalability of our approach to increasingly complex emotion taxonomies.

## 2. The Proposed CATE Architecture

### 2.1. Overall Architecture

**Figure 1:**
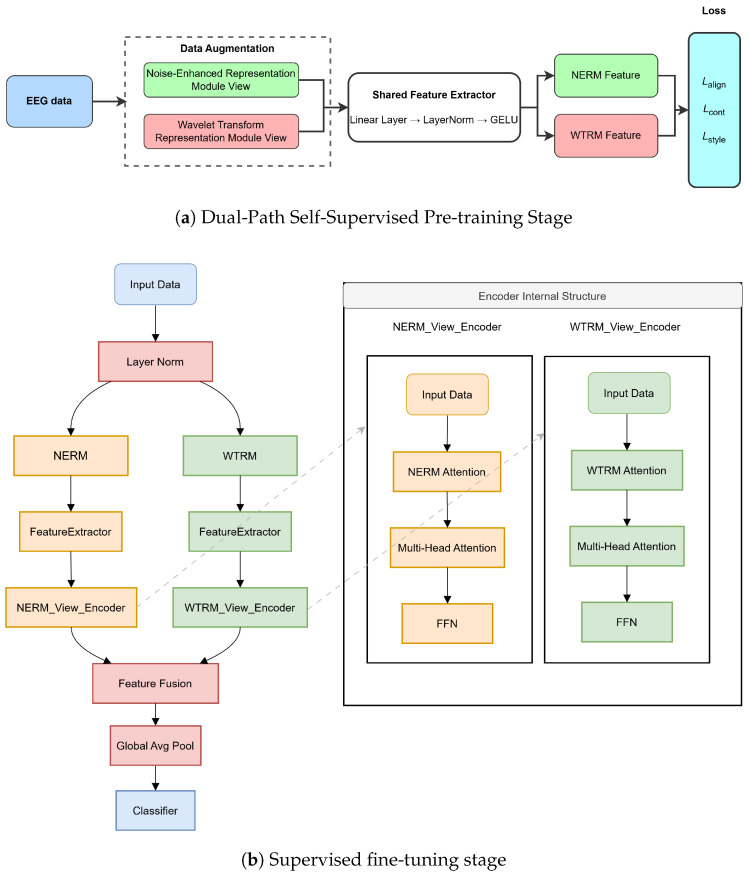
The overall architecture of the proposed CATE model for cross-corpus EEG-based emotion recognition. (**a**) Dual-Path Self-Supervised Pre-training Stage: The input EEG data undergoes dual-view data augmentation through Noise-Enhanced Representation Modeling (NERM) and Wavelet Transform Representation Modeling (WTRM). Both views are processed through a shared feature extractor with linear layers, LayerNorm, and GELU activation. The extracted NERM and WTRM features are then optimized using alignment loss ($L_{align}$), and style loss ($L_{style}$) to learn robust domain-invariant representations. (**b**) Supervised fine-tuning stage: The pre-trained model is adapted for emotion classification. Input data is normalized and processed through parallel NERM and WTRM view encoders, each containing attention mechanisms and feed-forward networks (FFN). The dual-view features are fused, pooled globally, and fed to a classifier for final emotion prediction. The inset shows the detailed internal structure of the encoders with multi-head attention layers.

The overall architecture of CATE is shown in Figure 1. It consists of two stages: (1) a dual-path attention-driven self-supervised pre-training stage based on frequency band perturbation view and wavelet modulation view, and (2) a supervised fine-tuning stage.

In the pre-training stage, the model learns robust representations from two complementary views through a shared feature extractor consisting of linear layers with LayerNorm and GELU activation. This simplified architecture focuses on learning domain-invariant features through contrastive learning across the NERM and WTRM views.

In the fine-tuning stage, the outputs of the pre-trained dual-path attention encoders are fused through attention-weighted feature integration and then connected to a task-specific classifier, enabling the model to effectively focus on key emotion-related patterns in EEG signals and achieve more robust cross-corpus emotion recognition.

The pre-training architecture creates two distinct augmented views of EEG data through specialized enhancement modules, followed by direct feature extraction for robust representation learning. The framework generates two complementary views from the original EEG differential entropy features to capture diverse signal characteristics.

### 2.2. The Dual-Path Self-Supervised Pre-Training Stage

(1) the shared feature extractor ensures both views learn in the same feature space; (2) the alignment loss ensures cross-view consistency by minimizing the distance between representations of the same sample across views; (3) the style diversity loss prevents representation collapse and ensures the two views learn complementary rather than redundant features.

#### 2.2.1. Noise-Enhanced Representation Modeling (NERM) View

Given the input EEG differential entropy features $X\in R^{B\times C\times F}$, where *B* denotes the batch size, *C* represents the number of channels, and F=5 indicates the five frequency bands (delta, theta, alpha, beta, gamma), the NERM view introduces a simple yet effective adaptive noise mechanism. This process enhances the robustness of the learned representations against domain-specific variations.

The core of this view is to add Gaussian noise whose magnitude is dynamically scaled based on the input features. The procedure is as follows:

First, an input scale is computed based on the mean absolute value of the features:

(1) $$s_{\mathrm{input}}=\mathrm{mean}\left(|X|\right)$$

Next, this input scale is used to determine the noise scale, which is capped to prevent excessive perturbation:

(2) $$s_{\mathrm{noise}}=\min\left(0.05,0.1\;\cdot \;s_{\mathrm{input}}+\epsilon \right)$$

where ϵ is a small constant (e.g., $1\times 10^{-8}$) to ensure numerical stability.

Finally, the noise-enhanced features $X_{\mathrm{NERM}}$ are generated by adding scaled Gaussian noise to the original features:

(3) $$X_{\mathrm{NERM}}=X+N\left(0,I\right)\;\cdot \;s_{\mathrm{noise}}$$

where N(0,I) represents standard Gaussian noise.

This adaptive noise injection strategy forces the model to learn features that are less sensitive to minor variations and artifacts present in the input. By dynamically adjusting the noise level relative to the input’s magnitude, it ensures that the perturbation is meaningful without corrupting the underlying signal structure. This process effectively regularizes the model, discouraging it from overfitting to spurious, domain-specific characteristics and thereby improving its generalization capability for cross-corpus emotion recognition tasks.

#### 2.2.2. Wavelet Transform Representation Modeling (WTRM) View

The WTRM view employs wavelet decomposition to capture multi-scale spectral energy distribution patterns across frequency bands:

For each sample $x_{i}\in R^{C\times F}$ where C=62 channels and F=5 frequency bands, the wavelet decomposition is applied to the spectral energy profile of each channel:

(4) $$\left\{c_{A}^{\left(c\right)},{\left\{c_{D_{j}}^{\left(c\right)}\right\}}_{j=1}^{L}\right\}=\mathrm{DWT}\left(x_{i}^{\left(c\right)},\psi ,L\right),\;c=1,\ldots ,C$$

where $x_{i}^{\left(c\right)}={\left[DE_{delta},DE_{theta},DE_{alpha},DE_{beta},DE_{gamma}\right]}^{\left(c\right)}$ represents the differential entropy features across five frequency bands for channel *c*, $c_{A}^{\left(c\right)}$ captures the overall spectral energy trend, $\left\{c_{D_{j}}^{\left(c\right)}\right\}$ captures the inter-band energy variations, ψ is the wavelet basis function (Haar or db1 due to signal length constraint), and L=1 is the decomposition level (limited by F=5).

Enhanced coefficients are generated through adaptive masking and perturbation:

(5) $${\tilde{c}}_{A}^{\left(c\right)}=c_{A}^{\left(c\right)}⊙\left(1+\mu_{A}\;\cdot \;M_{A}\;\cdot \;\epsilon_{A}\right)$$

(6) $${\tilde{c}}_{D_{j}}^{\left(c\right)}=c_{D_{j}}^{\left(c\right)}⊙\left(0.5\;\cdot \;M_{suppress}+M_{enhance}\;\cdot \;\left(1+\epsilon_{D_{j}}\;\cdot \;\sigma_{D_{j}}\right)\right)$$

where $M_{A},M_{suppress},M_{enhance}$ are random binary masks with probabilities μ×0.5, μ, and μ×0.5, respectively, μ=0.05 is the mask probability, $\epsilon_{A},\epsilon_{D_{j}}$ are enhancement factors, and ⊙ denotes element-wise multiplication.

The WTRM view is reconstructed via an inverse wavelet transform:

(7) $$X_{WTRM}^{\left(c\right)}=\mathrm{IDWT}\left(\left\{{\tilde{c}}_{A}^{\left(c\right)},{\left\{{\tilde{c}}_{D_{j}}^{\left(c\right)}\right\}}_{j=1}^{L}\right\},\psi \right)$$

This decomposition captures the spectral energy distribution patterns that are characteristic of different emotional states, where the approximation coefficients represent the overall brain activation level and the detail coefficients capture the coordination between adjacent frequency bands, providing complementary information to the NERM view for robust emotion recognition.

#### 2.2.3. Shared Feature Extraction

Both augmented views are processed through a shared feature extractor that directly maps the input to the latent representation space:

(8) $$H_{base}=\mathrm{LayerNorm}\left(\mathrm{GELU}\left(W_{1}X+b_{1}\right)\right)W_{2}+b_{2}$$

where $W_{1}\in R^{d_{model}\times d_{input}}$ and $W_{2}\in R^{d_{model}\times d_{model}}$ are learnable weight matrices, and $b_{1},b_{2}$ are bias vectors.

The features from both views are directly obtained through the shared extractor:

(9) $$Z_{NERM}=H_{base}\left(X_{NERM}\right)$$

(10) $$Z_{WTRM}=H_{base}\left(X_{WTRM}\right)$$

where $Z_{NERM},Z_{WTRM}\in R^{B\times d_{model}}$ represent the extracted features from NERM and WTRM views, respectively.

#### 2.2.4. Multi-Component Loss Function

The self-supervised pre-training optimizes three complementary loss components operating on the directly extracted features:

(1) Alignment Loss: This component ensures cross-view consistency by aligning representations from the same sample across different views in the feature space:
  (11) $$L_{align}=2-2\;\cdot \;\frac{1}{B}\sum_{i=1}^{B}\frac{z_{NERM}^{\left(i\right)}\;\cdot \;z_{WTRM}^{\left(i\right)}}{∥z_{NERM}^{\left(i\right)}{∥}_{2}{∥z_{WTRM}^{\left(i\right)}∥}_{2}}$$
  where $z_{NERM}^{\left(i\right)}$ and $z_{WTRM}^{\left(i\right)}$ represent L2-normalized features from NERM and WTRM views, respectively. This loss function promotes semantic consistency between complementary views while preserving view-specific characteristics.
(2) Style Diversity Loss: This component prevents representation collapse by enforcing orthogonality between different samples within the same view:
  (12) $$L_{style}=∥G_{NERM}{-I∥}_{F}^{2}+{∥G_{WTRM}-I∥}_{F}^{2}$$
  (13) $$G_{NERM}=Z_{NERM}Z_{NERM}^{T},\;G_{WTRM}=Z_{WTRM}Z_{WTRM}^{T}$$
  I is the identity matrix, and ${∥\;\cdot \;∥}_{F}$ denotes the Frobenius norm. By constraining the Gram matrices to approximate the identity matrix, this loss effectively maintains feature diversity and prevents dimensional collapse during training.
(3) Total Pre-training Loss: The complete pre-training objective combines the alignment and diversity components with appropriate weighting:
  (14) $$L_{total}=\lambda_{align}L_{align}+\lambda_{style}L_{style}$$
  where $\lambda_{align}$ and $\lambda_{style}$ are balancing coefficients that control the relative importance of alignment and diversity objectives. The combination of these two loss components forms the complete optimization objective for our self-supervised pre-training stage.

Through this multi-component loss framework, NERM and WTRM views form a synergistic interaction during pre-training: NERM focuses on learning representations robust to domain-specific noise and artifacts, while WTRM captures multi-scale spectral energy distribution patterns across frequency bands through wavelet decomposition. The alignment loss ensures both views produce consistent high-level semantic understanding of the same emotional state, while the style loss maintains feature diversity, preventing the model from simply learning identical representations. This dual-view approach enables the model to capture both noise-robust features and emotion-relevant spectral characteristics simultaneously.

### 2.3. Supervised Fine-Tuning Stage

During the supervised fine-tuning stage, the pre-trained Shared Feature Extractor serves as the backbone. Its learned weights are used to initialize two parallel encoders: the NERM View Encoder and the WTRM View Encoder. Each encoder is then augmented with its own multi-head attention mechanism, allowing the model to adapt the powerful, pre-trained representations for the specific downstream classification task.

**Figure 2:**
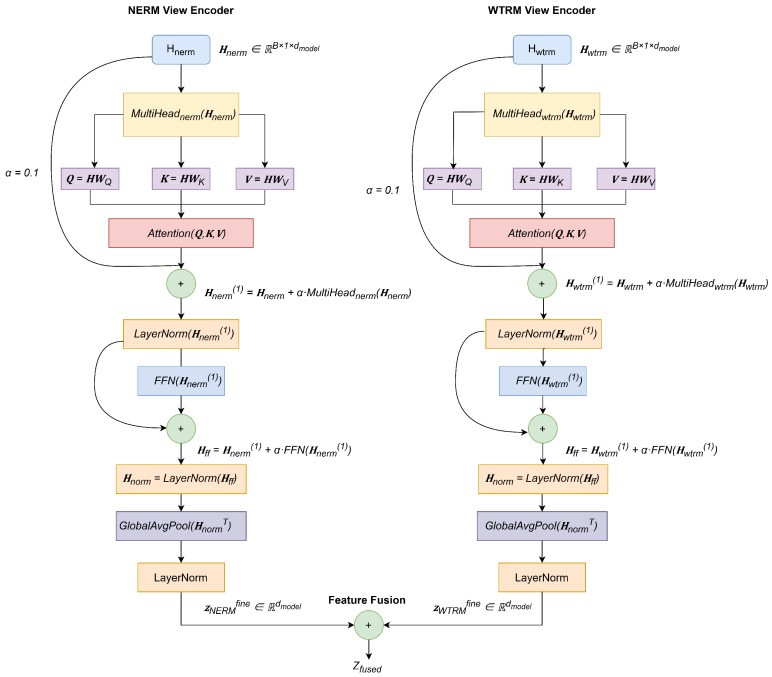
NERM-WTRM dual-view feature fusion architecture. Two complementary encoders process EEG representations from different perspectives: (1) The NERM encoder builds robustness against domain-specific artifacts through noise-enhanced processing and ${\mathrm{MultiHead}}_{nerm}$ attention; (2) The WTRM encoder extracts emotion-relevant spectral energy distributions through wavelet decomposition and ${\mathrm{MultiHead}}_{wtrm}$ attention. The complementary features $z_{NERM}^{fine}$ and $z_{WTRM}^{fine}$ are fused to form the robust joint representation $z_{fused}$ for emotion classification.

The model input consists of standard Differential Entropy (DE) features, extracted from EEG signals that have been downsampled to 200 Hz and band-pass filtered (1–75 Hz). To adapt these sample-based DE features for our Transformer-based architecture, we treat each vector as a sequence of length one (L=1). An input batch of shape $\left[B,d_{\mathrm{input}}\right]$ is reshaped to $\left[B,1,d_{\mathrm{input}}\right]$ and processed through the initialized encoders. As illustrated in Figure 2, the refined features from the parallel NERM and WTRM encoders are then fused to generate a joint representation for classification. This strategy allows the model to effectively leverage the powerful pre-trained architecture with non-sequential input data.

This strategy allows the model to effectively leverage the powerful pre-trained architecture, even with input data that is not inherently sequential. The proposed dual-view encoder architecture is illustrated in Figure 2. This architecture employs two parallel encoders, NERM and WTRM, to process EEG feature representations from different domains, ultimately generating a joint representation through feature fusion for emotion classification.

#### 2.3.1. Multi-Head Attention Mechanism

The core multi-head attention mechanism operates on the pre-trained features through learned query, key, and value projections:

(15) $$Q=HW_{Q},\;K=HW_{K},\;V=HW_{V}$$

where $H\in R^{B\times L\times d_{\mathrm{model}}}$ (with L=1 in our case) represents the input feature sequence, and $W_{Q},W_{K},W_{V}\in R^{d_{\mathrm{model}}\times d_{\mathrm{model}}}$ are learnable projection matrices.

The scaled dot-product attention is computed as:

(16) $$\mathrm{Attention}\left(Q,K,V\right)=\mathrm{softmax}\left(\frac{QK^{T}}{\sqrt{d_{k}}}\right)V$$

where $d_{k}=d_{\mathrm{model}}/h$ and *h* denotes the number of attention heads.

Multi-head attention concatenates outputs from multiple attention heads:

(17) $$\mathrm{MultiHead}\left(Q,K,V\right)=\mathrm{Concat}\left({\mathrm{head}}_{1},\ldots ,{\mathrm{head}}_{h}\right)W_{O}$$

where ${\mathrm{head}}_{i}=\mathrm{Attention}\left(QW_{Q}^{i},KW_{K}^{i},VW_{V}^{i}\right)$ and $W_{O}\in R^{d_{\mathrm{model}}\times d_{\mathrm{model}}}$ is the output projection matrix.

#### 2.3.2. NERM View Encoder

The NERM view adaptive encoder processes noise-enhanced representations through dual attention mechanisms to capture frequency-aware patterns:

(18) $$H_{nerm}^{\left(1\right)}=H_{nerm}+\alpha \;\cdot \;{\mathrm{MultiHead}}_{nerm}\left(H_{nerm}\right)$$

(19) $$H_{nerm}^{\left(1\right)}=\mathrm{LayerNorm}\left(H_{nerm}^{\left(1\right)}\right)$$

The final NERM representation is obtained through global average pooling:

(20) $$z_{NERM}^{fine}=\mathrm{LayerNorm}\left(\mathrm{GlobalAvgPool}\left(H_{nerm}^{\left(1\right)}\right)\right)$$

#### 2.3.3. WTRM View Encoder

The WTRM view encoder employs dual attention mechanisms to capture spectral energy distribution patterns from wavelet-enhanced representations.

(21) $$H_{wtrm}^{\left(1\right)}=H_{wtrm}+\alpha \;\cdot \;{\mathrm{MultiHead}}_{wtrm}\left(H_{wtrm}\right)$$

where α=0.1 is a scaling factor for residual connections to ensure training stability.

The combined representation is normalized:

(22) $$H_{wtrm}^{\left(1\right)}=\mathrm{LayerNorm}\left(H_{wtrm}^{\left(1\right)}\right)$$

The representation is further refined through a feed-forward network with scaled residual connection:

(23) $$H_{ff}=H_{wtrm}^{\left(1\right)}+\alpha \;\cdot \;\mathrm{FFN}\left(H_{wtrm}^{\left(1\right)}\right)$$

where FFN consists of:

(24) $$\mathrm{FFN}\left(x\right)=\mathrm{Dropout}(W_{2}\;\cdot \;\mathrm{GELU}\left(\mathrm{Dropout}\left(W_{1}\;\cdot \;x\right)\right))$$

with $W_{1}\in R^{d_{model}\times 4d_{model}}$ and $W_{2}\in R^{4d_{model}\times d_{model}}$.

After layer normalization:

(25) $$H_{norm}=\mathrm{LayerNorm}\left(H_{ff}\right)$$

The final WTRM representation for fine-tuning is obtained through adaptive pooling:

(26) $$z_{WTRM}^{fine}=\mathrm{LayerNorm}\left(\mathrm{GlobalAvgPool}\left(H_{norm}^{T}\right)\right)$$

#### 2.3.4. Cross-Domain Feature Fusion

The enhanced features from the NERM and WTRM views, $z_{NERM}^{fine}$ and $z_{WTRM}^{fine}$, are combined to form a final representation. While complex fusion strategies like attention-weighting are possible, we opted for simple averaging:

(27) $$z_{fused}=\frac{z_{NERM}^{fine}+z_{WTRM}^{fine}}{2}$$

This choice was based on a combination of empirical evidence, stability, and simplicity. As detailed in Appendix B, we conducted experiments on the SEED-${\mathrm{IV}}^{4}\to$ SEED-$V^{4}$ task to evaluate different fixed weighting ratios (see Table A5).

#### 2.3.5. Feature Fusion and Classification

Classification is performed by a multi-layer perceptron (MLP) head that takes the fused features $z_{\mathrm{fused}}$ as input. The MLP projects the features into a hidden representation, applies a GELU activation and dropout for regularization, and finally produces the output logits for classification.

(28) $$h_{\mathrm{cls}}=\mathrm{GELU}\left(\mathrm{LayerNorm}\left(W_{1}z_{\mathrm{fused}}+b_{1}\right)\right)$$

(29) $$h_{\mathrm{cls}}=\mathrm{Dropout}\left(h_{\mathrm{cls}}\right)$$

(30) $$\mathrm{logits}=W_{2}h_{\mathrm{cls}}+b_{2}$$

where $W_{1}\in R^{d_{\mathrm{hidden}}\times d_{\mathrm{model}}}$ and $W_{2}\in R^{d_{\mathrm{output}}\times d_{\mathrm{hidden}}}$ are learnable weight matrices.

#### 2.3.6. Fine-Tuning Objective

The fine-tuning stage adapts the model to the downstream classification task by optimizing a supervised objective. The loss function, therefore, consists of a standard cross-entropy classification loss:

(31) $$L_{\mathrm{fine}-\mathrm{tune}}=L_{\mathrm{cls}}$$

The classification loss is defined as the cross-entropy between the predicted logits and the one-hot encoded true labels:

(32) $$L_{\mathrm{cls}}=\mathrm{CrossEntropy}\left(\mathrm{logits},y_{\mathrm{true}}\right)$$

#### 2.3.7. Training Strategy

The fine-tuning process employs several standard optimization strategies to ensure stable and efficient training:

(1) Mixed Precision Training: We employ automatic mixed precision (AMP) with gradient scaling to accelerate the fine-tuning process while maintaining numerical stability.
(2) Gradient Clipping: To prevent the issue of exploding gradients, the norms of the gradients are clipped to a maximum threshold of 1.0, calculated as:
  (33) $$g_{\mathrm{clipped}}=\min\left(1.0,\frac{1.0}{{∥g∥}_{2}}\right)\;\cdot \;g$$
(3) Learning Rate Scheduling: A ReduceLROnPlateau scheduler is utilized with a factor of 0.5 and a patience of 5 epochs. This scheduler monitors the total loss and adaptively adjusts the learning rate when improvements stagnate.

This fine-tuning strategy allows the model to effectively adapt the learned representations for the emotion classification task. The combination of a direct classification objective with robust optimization techniques ensures that the model is efficiently tailored to the downstream task.

## 3. Experimental Setting

### 3.1. Datasets

The SEED series of datasets is an emotional electroencephalogram (EEG) database established by the BCMI Laboratory at Shanghai Jiao Tong University. This series includes three main datasets, each targeting classification for a different number of emotional categories.

The SEED dataset (original version) focuses on three-category emotion recognition (negative, neutral, positive). It involved 15 subjects participating in 3 experimental sessions, each watching 15 emotion-inducing film clips. EEG signals were recorded using a 62-channel ESI Neuroscan system, and various EEG features such as differential entropy (DE) and differential asymmetry (DASM) were extracted.

The SEED-IV dataset expands to four-category classification (neutral, sad, fear, and happy emotions). It also involved 15 subjects participating in 3 experimental sessions, but each session included 24 film clips.

The SEED-V dataset further expands to five-category classification (disgust, fear, sad, neutral, happy). Each subject participated in 3 experimental sessions, watching 15 film clips per session, with both EEG and eye-tracking data recorded.

### 3.2. Pre-Processing

After collecting the raw EEG data, signal preprocessing and feature extraction performed. To increase the signal-to-noise ratio (SNR), the raw EEG signals are first downsampled to a 200 Hz sampling rate, followed by a band-pass filter from 1 Hz to 75 Hz. Subsequently, feature extraction is carried out. Differential Entropy (DE), as referenced in the literature [18], has the ability to distinguish patterns in different frequency bands. Therefore, we chose DE features as the input data for our model.

For one subject in one session in these three databases, a set of data is given in the form of channels (62) × trials (15 for SEED, 24 for SEED-IV, 15 for SEED-V) × frequency bands (5). We merge the channels and frequency bands, changing the form to trials × 310 (62×5).

Finally, all data are organized into 3394×310 (SEED dataset,3394 samples per subject per session), or 851/832/822×310 (SEED-IV dataset,851/832/822 samples per subject per session, respectively), or 681/541/601×310 (SEED-V dataset,681/541/ 601 samples per subject per session, respectively). The corresponding label vectors are generated in the form of 3394×1, or 851/832/822×1, or 681/541/601×1.

### 3.3. Evaluation Metrics

Accuracy is used as a measure to evaluate the EEG classification, which is calculated as follows:

(34) $$\mathrm{acc}=\frac{A}{\mathrm{Num}}$$

where *A* refers to the number of samples correctly classified by the algorithm, and Num refers to the total number of samples to be classified.

## 4. Experiments

### 4.1. Experimental Setup

#### 4.1.1. Datasets and Baselines

We evaluated our proposed model, CATE, on three publicly available EEG emotion recognition datasets: SEED [19], SEED-IV [20], and SEED-V [21]. We conducted a comprehensive comparison against five state-of-the-art (SOTA) methods: DANN [22], GECNN [23], E2STN [24], JCFA [17], and Mixup [14]. For fair comparison, all baselines were implemented using their officially reported hyperparameters.

#### 4.1.2. Evaluation Protocol

We followed a strict subject-independent, cross-corpus protocol with supervised target-domain fine-tuning applied consistently across all methods. Importantly, all baseline models and our proposed CATE method were evaluated under the identical protocol, where target domain labels were used during fine-tuning. The experiments were conducted across six transfer tasks between the datasets. We use accuracy (ACC) and its standard deviation across all subjects in the test set as the primary evaluation metric. The specific trial splits for fine-tuning and testing in each task were as follows:

- SEED ↔ SEED-${\mathrm{IV}}^{3}$: For SEED, 9 trials were used for fine-tuning and 6 for testing. For SEED-IV (3-class), 12 trials were used for fine-tuning and 6 for testing.
- SEED ↔ SEED-$V^{3}$: For SEED, 9 trials for fine-tuning, 6 for testing. For SEED-V (3-class subset), 6 trials for fine-tuning, 3 for testing.
- SEED-${\mathrm{IV}}^{4}$↔ SEED-$V^{4}$: For SEED-IV (4-class), 16 trials for fine-tuning, 8 for testing. For SEED-V (4-class subset), 8 trials for fine-tuning, 4 for testing.

#### 4.1.3. Implementation Details

Models were implemented in Python 3.8 using PyTorch 1.9.0 and trained on an NVIDIA Tesla A10 GPU. The self-supervised pre-training was conducted for 200 epochs with a batch size of 256. The subsequent fine-tuning was performed for 20 epochs with a batch size of 128.

### 4.2. Cross-Corpus Performance Analysis

Table 2 presents the performance comparison across all six cross-corpus tasks. Our proposed CATE model consistently and significantly outperforms all baseline methods, establishing a new state-of-the-art. Notably, CATE demonstrates not only higher mean accuracy but also lower standard deviation in most cases, indicating superior performance and stability.

For instance, in the SEED → SEED-${\mathrm{IV}}^{3}$ and SEED-${\mathrm{IV}}^{3}$→ SEED tasks, CATE achieves accuracies of 80.72% and 80.70%, surpassing the second-best method, JCFA, by a large margin of 15.20% and 14.37%, respectively. This substantial improvement highlights the effectiveness of our dual-view pre-training strategy. The Noise-Enhanced Representation Modeling (NERM) view learns to discard domain-specific noise stemming from different acquisition hardware, while the Wavelet Transform Representation Modeling (WTRM) view captures domain-invariant, multi-scale temporal-frequency patterns crucial for emotion recognition. The synergy between these views enables the model to learn robust and generalizable EEG representations.

The performance gains are also evident in the more challenging tasks involving SEED-V, which features a more complex emotion taxonomy. In the SEED-$V^{4}$→ SEED-${\mathrm{IV}}^{4}$ task, CATE achieves a 13.41% improvement over JCFA. This scenario, involving transfer between two distinct 4-class paradigms, underscores CATE’s ability to handle complex domain shifts and partially overlapping emotion categories.

**Figure 3:**
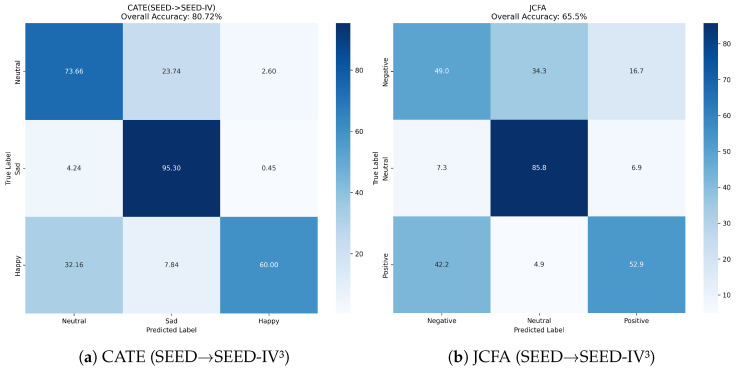

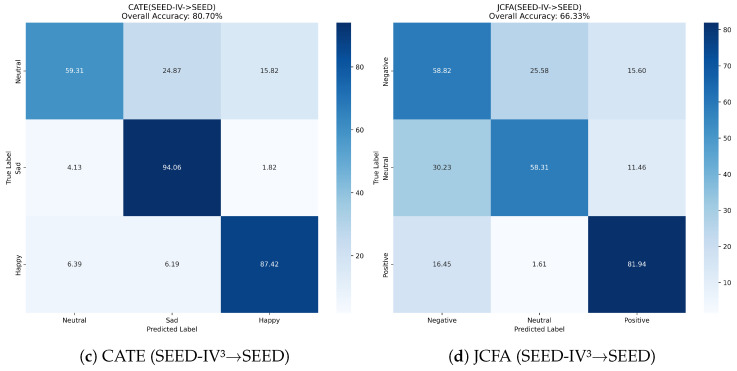
Confusion matrices for CATE vs. JCFA on the SEED ↔ SEED-IV^3^ tasks.

**Figure 4:**
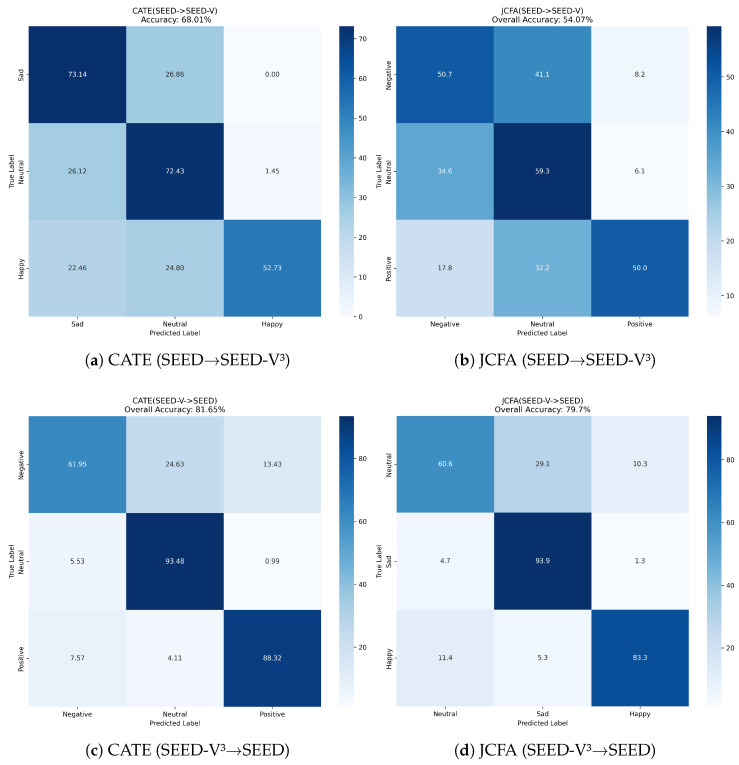
Confusion matrices for CATE vs. JCFA on the SEED ↔ SEED-V^3^ tasks.

**Figure 5:**
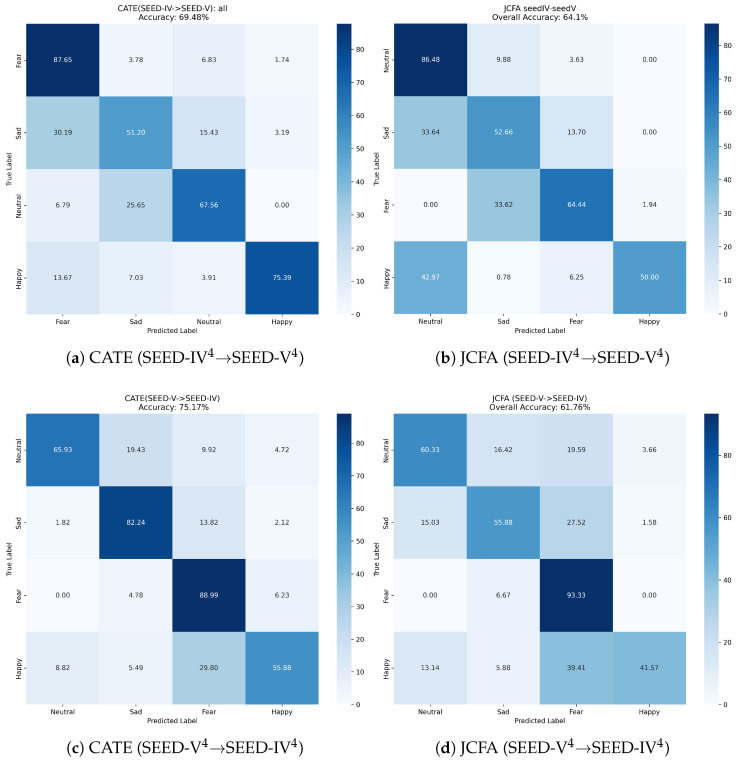
Confusion matrices for CATE vs. JCFA on the SEED-${\mathrm{IV}}^{4}$↔ SEED-$V^{4}$ tasks.

Further analysis of the confusion matrices (Figure Figure 3, Figure 4 and Figure 5) reveals CATE’s discriminative power. For example, in the SEED → SEED-${\mathrm{IV}}^{3}$ task (Figure a), CATE significantly reduces the confusion between ‘positive’ and ‘neutral’ emotions compared to JCFA (Figure b). Similarly, in the challenging SEED-${\mathrm{IV}}^{4}$→ SEED-$V^{4}$ experiment (Figure 5c), CATE improves the recognition of ‘fear’ and reduces its confusion with ‘sad’, suggesting its capability to distinguish between nuanced emotional states. This confirms that our model learns more discriminative features, rather than just overfitting to the source domain’s characteristics.

### 4.3. Ablation Study

To validate the contributions of the core components of CATE, we conducted an extensive ablation study across all six tasks. We designed three variants: (1) w/o NERM, which removes the noise-enhanced view; (2) w/o WTRM, which removes the wavelet-based view; and (3) w/o pretrain, which trains the full model from scratch without self-supervised pre-training.

The results are summarized in Table 3. The full CATE model achieves the best performance across all settings, demonstrating the efficacy of its integrated design.

- Removing the NERM component (‘w/o NERM’) causes the most significant performance degradation across all tasks (e.g., a 7.06% drop in the SEED → SEED-${\mathrm{IV}}^{3}$ task). This confirms that modeling and mitigating domain-specific noise is critical for cross-corpus generalization.
- Removing the WTRM component (‘w/o WTRM’) also leads to a notable performance decline, highlighting the importance of capturing multi-scale temporal-frequency features, which serve as robust, emotion-related biomarkers.
- Training the model from scratch (‘w/o pretrain’) results in inferior performance compared to the full model. This validates that the dual-view self-supervised pre-training provides a powerful initialization, enabling the model to learn more transferable representations before fine-tuning on limited labeled data.

**Table 3:** Ablation study results: average accuracy and standard deviation (%) of different model configurations across all cross-dataset tasks.

Method	SEED → SEED-${\mathrm{IV}}^{3}$	SEED-${\mathrm{IV}}^{3}$ → SEED	SEED → SEED-$V^{3}$	SEED-$V^{3}$ → SEED	SEED-${\mathrm{IV}}^{4}$ → SEED-$V^{4}$	SEED-$V^{4}$ → SEED-${\mathrm{IV}}^{4}$
w/o NERM	73.66 ± 18.84	74.50 ± 17.83	52.36 ± 23.52	79.85 ± 12.60	66.53 ± 20.76	70.51 ± 16.47
w/o WTRM	73.82 ± 19.13	78.99 ± 18.32	50.37 ± 20.48	74.61 ± 14.23	64.44 ± 15.53	66.62 ± 14.83
w/o pretrain	77.52 ± 18.69	76.25 ± 16.47	62.36 ± 19.82	79.05 ± 10.23	62.40 ± 19.51	71.86 ± 15.94
**Full (CATE)**	**80.72 ± 14.27**	**80.70 ± 10.09**	**68.01 ± 19.33**	**81.65 ± 09.35**	**69.48 ± 16.77**	**75.17 ± 14.01**

The results demonstrate the contribution of each key component in the CATE model. NERM: Noise-Enhanced Representation Modeling; WTRM: Wavelet Transform Representation Modeling.

In summary, the ablation study empirically proves that the superior performance of CATE stems from the powerful synergy between its noise-robust modeling (NERM), multi-scale feature extraction (WTRM), and the effective feature initialization provided by the self-supervised pre-training paradigm.

## 5. Discussion

The empirical results robustly demonstrate that CATE sets a new state-of-the-art in cross-corpus EEG emotion recognition. Our discussion focuses on interpreting these performance gains by linking them to our model’s core architectural innovations, contextualizing its advantages over existing methods, and outlining the broader implications of this work.

### 5.1. Interpretation of CATE’s Performance

The consistent and substantial outperformance of CATE across all six transfer tasks validates our central hypothesis: a dual-view, self-supervised pre-training framework can effectively learn domain-invariant representations from heterogeneous EEG data. The most significant accuracy improvements (over 14 percentage points against the next-best method in SEED ↔ SEED-IV^3^ tasks) are not merely incremental gains but signify a fundamental advantage in generalization capability.

This advantage stems directly from the synergistic interplay of our two complementary views. The Noise-Enhanced Representation Modeling (NERM) view forces the model to become robust against superficial, domain-specific signal characteristics—akin to learning to recognize a voice despite different background noises or microphone qualities. This is crucial for cross-corpus tasks where recording equipment and environments inevitably vary. Concurrently, the Wavelet Transform Representation Modeling (WTRM) view captures the intrinsic, multi-scale temporal-frequency dynamics that are foundational to emotional expression in EEG signals. The ablation study (Table 3) provides clear evidence for this synergy: removing either view results in a significant performance drop, with the removal of NERM being particularly detrimental, confirming that tackling domain noise is a primary challenge.

Furthermore, the multi-head attention mechanism in the fine-tuning stage proves essential for adapting these robustly pre-trained features to specific classification tasks. As seen in the confusion matrices (e.g., Figure 5), CATE excels at distinguishing between nuanced and often-confused emotional states like ‘sad’ and ‘fear’. This suggests that the attention mechanism effectively learns to focus on the subtle yet discriminative neural signatures captured by the dual-view encoders, a capability that simpler feature extractors may lack.

### 5.2. Visualization Analysis of Feature Representations

**Figure 6:**
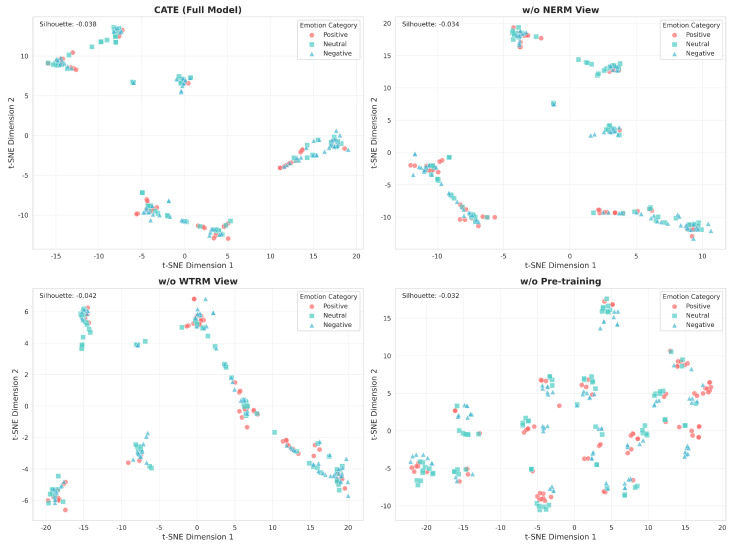
t-SNE visualization of learned feature representations from different CATE model configurations on the SEED dataset. Each point represents a sample projected into 2D space, colored by emotion category (red: positive, green: neutral, blue: negative).

To gain deeper insights into how each component of CATE contributes to effective emotion representation learning, we conducted t-SNE visualization analysis of the learned features across different model configurations. We randomly selected 200 samples from the SEED dataset and employed t-SNE to project the high-dimensional feature representations into a two-dimensional space. Figure 6 presents the t-SNE projections of feature representations from four model variants, providing intuitive evidence of each component’s contribution to discriminative feature learning.

From the perspective of an ablation study, the visualization results reveal several critical insights:

First, regarding the relative advantages of the full CATE model: Among the four configurations, the complete model demonstrates the relatively best feature organization structure. While overlaps between the three emotion categories still exist (which is common in EEG-based emotion recognition), compared to other configurations, the complete model exhibits more regular feature point distribution with relatively clearer inter-class boundaries. This indicates that the synergistic effect of the dual-view architecture and pre-training strategy indeed contributes to learning more discriminative feature representations.

Second, regarding the contribution of the NERM view encoder: When the NERM component is removed, feature distribution significantly deteriorates, manifesting as increased class overlap, particularly between neutral and negative emotions. This distribution degradation validates our quantitative findings that NERM view’s noise-enhanced modeling is crucial for extracting robust features that can distinguish subtle emotional differences in the presence of domain-specific noise. The absence of this component leads to the model’s difficulty in discriminating fine-grained emotional variations.

Third, regarding the effectiveness of the WTRM view encoder: Removing the WTRM component also compromises feature discriminability, though to a slightly lesser extent than removing NERM. The clusters show increased dispersion and partial overlap, particularly in the boundary regions between emotions. This suggests that wavelet-based multi-scale temporal-frequency features provide complementary information essential for capturing the full spectrum of emotion-related neural dynamics.

Most significantly, regarding the importance of the pre-training strategy: Compared to the full model, the model without pre-training exhibits the poorest feature organization, with all three emotion categories heavily intermingled. Despite achieving reasonable classification accuracy through supervised fine-tuning alone, the lack of self-supervised pre-training results in features that fail to capture the underlying structure of emotional states. This powerfully demonstrates that our dual-view pre-training strategy learns fundamental representations that supervised learning alone cannot achieve.

Core findings from the ablation study: The comprehensive ablation analysis reveals distinct contributions from each component across different transfer scenarios:

(1) Component-wise Analysis: Removing either the NERM or WTRM view consistently degrades performance, but their relative importance varies across tasks. NERM shows more significant impact in challenging cross-corpus scenarios (e.g., 7.06% drop in SEED → SEED-${\mathrm{IV}}^{3}$), while WTRM’s contribution is more pronounced in tasks involving complex emotion taxonomies (e.g., notable degradation in SEED-V related transfers).

(2) Pre-training vs. Architecture: The comparison between component removal and pre-training ablation reveals an interesting pattern. While removing individual components causes consistent performance drops, the impact of removing pre-training varies significantly across tasks. In some cases (e.g., SEED → SEED-${\mathrm{IV}}^{3}$), the architectural components (NERM/WTRM) contribute more than pre-training, while in others (e.g., SEED → SEED-$V^{3}$), pre-training shows greater importance. This suggests that the value of self-supervised pre-training is task-dependent and complements rather than replaces good architectural design.

(3) Synergistic Integration: The full model consistently outperforms all ablated versions, confirming that the optimal performance emerges from the synergistic combination of dual-view architecture and self-supervised pre-training, rather than from any single component dominance.

The t-SNE analysis provides compelling visual evidence supporting our architectural design choices. The synergistic combination of NERM and WTRM views, enhanced through self-supervised pre-training, creates a feature space where emotions are naturally organized along meaningful dimensions. This inherent structure in the learned representations explains CATE’s superior cross-corpus generalization capability—by learning features that capture the essential characteristics of emotional states rather than dataset-specific patterns, the model can effectively transfer knowledge across different experimental paradigms and recording conditions.

### 5.3. Comparison with Previous Cross-Corpus Methods

#### 5.3.1. Comparison with Traditional Domain Adaptation Methods

DANN (2016), as a classical domain adversarial method, attempts to align feature distributions between source and target domains through adversarial training. However, our experimental results show that DANN performs poorly across all tasks (e.g., only 45.20% on SEED → SEED-${\mathrm{IV}}^{3}$), which is 35.52 percentage points lower than CATE. This substantial gap exposes DANN’s fundamental limitation: its assumption that source and target distributions can be directly matched is brittle for highly disparate EEG domains. In contrast, CATE does not force explicit alignment but rather learns core representations that are inherently robust to domain shifts, proving to be a more effective strategy.

GECNN (2021) introduced graph-embedded convolution to model spatial relationships between EEG channels. While achieving 57.25% accuracy on SEED → SEED-${\mathrm{IV}}^{3}$, an improvement over DANN, it still falls 23.47 percentage points below CATE.GECNN’s primary limitation is its reliance on fixed spatial patterns that may not generalize across different recording setups and protocols. Without explicit mechanisms for handling domain-specific artifacts or capturing multi-scale spectral dynamics, GECNN struggles with the inherent variability in cross-corpus scenarios. Our dual-view architecture addresses these gaps through complementary noise-resilient (NERM) and wavelet-based spectral (WTRM) representations, achieving superior domain adaptation.

#### 5.3.2. Comparison with Recent Self-Supervised Methods

E2STN (2025) employs an emotion style transfer network, achieving 61.24% on SEED → SEED-${\mathrm{IV}}^{3}$. Despite attempting to mitigate domain differences through style transfer, it still lags behind CATE by 19.48 percentage points. E2STN’s main issue is that its style transfer mechanism assumes emotional “style” can be simply separated from content, but emotional expression in EEG signals is often tightly coupled with multiple aspects of the signal.

JCFA (2024) represents the closest competitor to CATE, employing dual-view contrastive learning with graph convolution. However, even this state-of-the-art method only achieves 65.52% on SEED → SEED-${\mathrm{IV}}^{3}$, 15.20 percentage points lower than CATE. More importantly, JCFA’s training time is prohibitively long due to the computational complexity of graph operations. Another critical limitation of JCFA is its lack of explicit noise handling mechanisms, while our NERM view is specifically designed to filter domain-specific artifacts.

### 5.4. Limitations and Future Work

Despite its strong performance, this study has limitations that open avenues for future research. First, our validation was conducted on the SEED family of datasets. While these are standard benchmarks, testing CATE’s generalization capabilities on a wider array of datasets with even greater diversity (e.g., DEAP, AMIGOS) is a necessary next step. Second, the current CATE model operates in an offline setting. For many real-world applications, such as real-time adaptive interfaces or clinical monitoring, online adaptation capabilities are required.

Future work will, therefore, focus on three key directions: (1) Model Lightweighting: We will explore model compression and knowledge distillation techniques to create a more efficient version of CATE suitable for deployment on edge devices. (2) Broader-Scale Validation: We will extend our evaluation to more diverse and larger-scale EEG datasets to further probe the limits of CATE’s generalization. (3) Online and Multimodal Extension: We plan to develop an online adaptation mechanism for CATE and investigate the fusion of EEG data with other modalities (e.g., eye-tracking, ECG) to build even more robust and comprehensive emotion recognition systems.

## 6. Conclusions

In this paper, we introduced CATE, a novel two-stage deep learning framework designed to overcome the critical challenge of cross-corpus generalization in EEG-based emotion recognition. By synergistically combining a dual-view self-supervised pre-training strategy with an attention-based fine-tuning mechanism, CATE effectively learns robust, transferable representations from complex and noisy EEG signals. The core innovation lies in its complementary Noise-Enhanced (NERM) and Wavelet Transform (WTRM) modeling views, which equip the model to handle domain-specific noise while capturing essential temporal-frequency patterns of emotional states.

Extensive experiments across six challenging transfer tasks using the SEED, SEED-IV, and SEED-V datasets demonstrate the definitive superiority of our approach. CATE consistently set a new state-of-the-art, outperforming existing methods by substantial margins of up to 15.65 percentage points. These results, supported by a thorough ablation study, validate the architectural choices and confirm the efficacy of each component within the CATE framework. By significantly bridging the gap between single-dataset performance and real-world applicability, this work represents a meaningful advancement towards deploying practical and reliable EEG-based affective computing systems. CATE provides a robust and promising paradigm for future research in cross-domain physiological signal analysis. 

## Figures and Tables

**Table 1:** Limitations in Existing Cross-Corpus EEG Emotion Recognition Methods.

Approach Category	Key Idea	Limitations in Cross-Corpus Context
**Conventional Supervised Learning**	Train a model on a large, labeled source dataset.	Fails to generalize to new target domains due to overfitting; performance drops significantly.
**Traditional Domain Adaptation**	Align the feature distributions of source and target domains, often adversarially.	Assumes distributions are close, which is often not the case for EEG; susceptible to negative transfer if domains are too dissimilar.
**Attention-Based Models**	Adaptively weight EEG channels, time steps, or frequency bands.	Often operate on a single view of the data, failing to capture the interplay between spatial, temporal, and spectral patterns.
**Self-Supervised & Contrastive Learning**	Learn representations from unlabeled data by solving pretext tasks or contrasting augmented views.	Learning is highly dependent on the choice of data augmentation; may not sufficiently account for domain-specific noise and style variations.

**Table 2:** Comparison of average accuracy and standard deviation (%) of different methods across the six cross-corpus emotion recognition tasks.

Method	SEED → SEED-${\mathrm{IV}}^{3}$	SEED-${\mathrm{IV}}^{3}$ → SEED	SEED → SEED-$V^{3}$	SEED-$V^{3}$ → SEED	SEED-${\mathrm{IV}}^{4}$ → SEED-$V^{4}$	SEED-$V^{4}$ → SEED-${\mathrm{IV}}^{4}$
DANN(2016) *	45.20 ± 24.31	62.89 ± 08.67	46.53 ± 23.52	62.27 ± 12.60	36.91 ± 14.89	33.50 ± 17.35
GECNN(2021)	57.25 ± 07.53	58.02 ± 07.03	-	-	-	-
MIXUP(2018) *	60.23 ± 15.56	63.24 ± 16.47	51.11 ± 20.66	72.21 ± 14.23	53.25 ± 20.34	59.78 ± 18.36
E2STN(2025)	61.24 ± 15.14	60.51 ± 05.41	-	-	-	-
JCFA(2024) *	65.52 ± 15.92	66.33 ± 11.15	54.07 ± 23.26	79.73 ± 12.32	64.06 ± 23.22	61.76 ± 15.23
**CATE (ours)**	**80.72 ± 14.27**	**80.70 ± 10.09**	**68.01 ± 19.33**	**81.65 ± 09.35**	**69.48 ± 16.77**	**75.17 ± 14.01**

* Results obtained by our own implementation. “-” indicates results were not available in the original papers.

## Data Availability

The database used in this study is publicly available at the following websites, SEED Dataset: https://bcmi.sjtu.edu.cn/home/seed/seed.html (accessed on 1 March 2021); SEED-IV Dataset: https://bcmi.sjtu.edu.cn/home/seed/seed-iv.html (accessed on 1 March 2021); SEED-V Dataset: https://bcmi.sjtu.edu.cn/home/seed/seed-v.html (accessed on 1 March 2022).

## Abbreviations

The following abbreviations are used in this manuscript:

CATE	Cross-corpus Attention-based Transfer Enhancement network
EEG	Electroencephalography
NERM	Noise-Enhanced Representation Modeling
WTRM	Wavelet Transform Representation Modeling

## Appendix A. Implementation Details and Hyperparameter Settings

### Appendix A.1. Training Configuration

**Table A1:** Hyperparameter settings for the pre-training stage.

Parameter	Value	Description
Optimizer	AdamW	Adaptive moment estimation with weight decay
Base learning rate	$4\times 10^{-4}$	Initial learning rate for pre-training
Weight decay	$3\times 10^{-4}$	L2 regularization coefficient
Training epochs	200	Total number of pre-training epochs
Batch size	256	Number of samples per batch
Gradient clipping threshold	1.0	Maximum gradient norm
Scheduler decay factor	0.5	Learning rate reduction ratio
Scheduler patience	5	Epochs to wait for improvement

**Table A2:** Hyperparameter settings for the fine-tuning stage.

Parameter	Value	Description
Optimizer	AdamW	Adaptive moment estimation with weight decay
Base learning rate	$2\times 10^{-4}$	Initial learning rate for fine-tuning
Weight decay	$3\times 10^{-4}$	L2 regularization coefficient
Training epochs	20	Total number of fine-tuning epochs
Batch size	128	Number of samples per batch

### Appendix A.2. Loss Function Weights

**Table A3:** Multi-component loss function weight configuration.

Loss Component	Pre-Training	Fine-Tuning	Description
$\lambda_{align}$ (Alignment loss)	0.5	0.0	Cross-view consistency weight
$\lambda_{style}$ (Style diversity loss)	0.5	0.0	Feature diversity weight
$\lambda_{cls}$ (Classification loss)	-	1.0	Cross-entropy classification weight

### Appendix A.3. Model Architecture Parameters

**Table A4:** CATE model architecture configuration.

Parameter	Value	Description
Input dimension $d_{input}$	310	62 channels × 5 frequency bands
Model dimension $d_{model}$	200	Feature representation dimension
Hidden dimension $d_{hidden}$	256	MLP hidden layer size
Output dimension $d_{output}$	3/4/5 *	Number of emotion classes
Number of attention heads *h*	8	Multi-head attention heads
Number of Transformer layers	2	Encoder layers
FFN expansion factor	4	Feed-forward network expansion ratio
Residual scaling factor α	0.1	Scaling coefficient for training stability

* Adjusted according to different dataset tasks.

## Appendix B. Feature Fusion Ablation Study

To determine the optimal method for fusing features from the NERM and WTRM views, we conducted an ablation study on the SEED-${\mathrm{IV}}^{4}\to$ SEED-$V^{4}$ transfer task, experimenting with various fixed weighting ratios. The results, presented in Table A5, show that performance peaks around a 0.6:0.4 to 0.5:0.5 ratio. Specifically, simple averaging (a 0.5:0.5 ratio) achieved performance (e.g., Accuracy: 69.48%, F1-Score: 65.31%) that was statistically indistinguishable from the best-performing, slightly unbalanced ratio (0.6:0.4). Given the negligible performance difference, we selected simple averaging as it is a parameter-free, more stable, and less complex approach. This choice avoids introducing additional hyperparameters that might require separate tuning for each cross-corpus task, thereby enhancing the model’s generalizability and robustness.

**Table A5:** Performance comparison of different feature fusion weighting ratios for the NERM and WTRM views on the SEED-${\mathrm{IV}}^{4}\to$ SEED-$V^{4}$ task. The best performance for each metric is highlighted in bold.

Ratio NERM:WTRM	Accuracy	Precision	F1-Score	AUROC	AUPRC
0.9 : 0.1	0.6813 ± 0.1960	0.6643 ± 0.2240	0.6333 ± 0.2209	0.8636 ± 0.1535	0.7960 ± 0.1894
0.8 : 0.2	0.6830 ± 0.1761	0.6807 ± 0.2103	0.6337 ± 0.1993	0.8648 ± 0.1511	0.7952 ± 0.1881
0.7 : 0.3	0.6937 ± 0.1651	0.6821 ± 0.2115	0.6458 ± 0.1889	0.8684 ± 0.1502	0.8012 ± 0.1879
0.6 : 0.4	**0.6948 ± 0.1617**	0.6827 ± 0.2095	0.6496 ± 0.1859	**0.8708 ± 0.1465**	**0.8044 ± 0.1849**
0.5 : 0.5	**0.6948 ± 0.1677**	**0.6946 ± 0.1942**	**0.6531 ± 0.1910**	0.8699 ± 0.1454	0.8019 ± 0.1839
0.4 : 0.6	0.6913 ± 0.1720	0.6933 ± 0.1942	0.6508 ± 0.1956	0.8682 ± 0.1423	0.7972 ± 0.1814
0.3 : 0.7	0.6868 ± 0.1668	0.6879 ± 0.1908	0.6456 ± 0.1922	0.8656 ± 0.1409	0.7910 ± 0.1803
0.2 : 0.8	0.6792 ± 0.1658	0.6827 ± 0.1910	0.6357 ± 0.1924	0.8629 ± 0.1397	0.7856 ± 0.1799
0.1 : 0.9	0.6708 ± 0.1607	0.6752 ± 0.1891	0.6282 ± 0.1869	0.8611 ± 0.1359	0.7782 ± 0.1785
